# The effect of perceptual expectation on processing gain, attention and the perceptual decision bias in children and adolescents with Autism Spectrum Disorder (ASD)

**DOI:** 10.1038/s41598-022-25971-z

**Published:** 2022-12-15

**Authors:** Sara Boxhoorn, Magdalena Schütz, Andreas M. Mühlherr, Hannah Mössinger, Christina Luckhardt, Christine M. Freitag

**Affiliations:** Department of Child and Adolescent Psychiatry, Psychotherapy and Psychosomatics, University Hospital Frankfurt, Goethe University, Deutschordenstraße 50, 60487 Frankfurt am Main, Germany

**Keywords:** Cognitive neuroscience, Attention, Cognitive control, Decision, Perception

## Abstract

Perceptual expectations influence perception, attention and the perceptual decision bias during visuospatial orienting, which is impaired in individuals with Autism Spectrum Disorder (ASD). In this study, we investigated whether during visuospatial orienting, perceptual expectations in ASD differentially influence perception, attention and the perceptual decision bias relative to neurotypical controls (NT). Twenty-three children and adolescents with ASD and 23 NT completed a visuospatial orienting task, which compared the effect of a valid relative to an invalid perceptual expectation on target detection (cue validity effect). Group differences were calculated regarding the cue validity effect on neural correlates of processing gain (N1a amplitude) and attention (N1pc amplitude), the perceptual decision bias and mean reaction time (RT). In ASD relative to NT, findings showed a reduced processing gain for validly relative to invalidly cued targets and increased attentional response following invalidly relative to validly cued targets. Increased attention correlated with faster performance across groups. Increased processing correlated with a higher perceptual decision bias and faster mean RT in NT, but not in ASD. Results suggest that during visuospatial orienting, perceptual expectations in ASD may drive changes in sensory processing and stimulus-driven attention, which may differentially guide behavioural responses.

## Introduction

Autism Spectrum Disorder (ASD) is a neurodevelopmental disorder defined by impairments in social communication and social interaction, and restricted, repetitive patterns of behavior, interests or activities^1^. Beyond these impairments defined by diagnostic criteria for ASD, ASD may come along with differences in perceptual and attentional processes, such as enhanced pitch discrimination^2^ and slower visuospatial orienting of attention^3^. These differences in perceptual processes and visuospatial orienting of attention may underlie the behaviorally defined phenotype described by the diagnostic criteria for ASD^4,5^. During visuospatial orienting of attention, perceptual processes such as perceptual expectations based on prior knowledge play a crucial role. That is, perceptual expectations prioritize processing of certain sensory input based on their expected likelihood, (i.e. the probability of observing *x* for each possible environmental state *y*). Orienting attention either requires processing input that matches a perceptual expectation, or during re-orienting, processing input incongruent with a perceptual expectation. Perceptual expectations in individuals with ASD would be less precise^5^, possibly because prior perceptual expectations are more slowly updated with new, incoming sensory information^6,7^. Individuals with ASD may thus perceive unexpected sensory input as unexpected for longer time periods as compared to neurotypical individuals. During orienting and re-orienting attention to relatively unexpected stimuli, previous findings suggest atypical activity of the locus coeruleus-norepinephrine system (LC-NE) in ASD^8,9^, which regulates processing gain through NE release in the cortex^10^. Processing gain refers to the adaptive, amplified cortical response to some, but not other sensory input^11^. Thus, an atypical anticipation of uncertainty in ASD may impair the processing gain of incoming sensory input in the cortex during visuospatial orienting. Perceptual expectations additionally influence other processes during visuospatial orienting, such as attention^12^ and the perceptual decision bias^13^. Nevertheless, to the best of our knowledge, no previous study investigated whether perceptual expectations differentially influence these processes during visuospatial orienting in ASD. To better understand the underlying processes of atypical visuospatial orienting in ASD, the present study investigated how perceptual expectations in ASD influence processing gain, attention and the perceptual decision bias.

The effect of a perceptual expectation during visuospatial orienting is typically measured with Posner paradigms by *the cue validity effect.* In Posner-like tasks, participants are required to respond as quickly as possible to an upcoming visuospatial target. A cue may either predict the upcoming target correctly (valid condition) or incorrectly (invalid condition). Validly cued targets additionally appear more frequently than invalidly cued targets, which further accelerates target detection^14^. The cue validity effect is calculated as the improvement in performance (mean reaction time) due to a valid relative to an invalid expectation. Feldman and Friston^12^ suggested processing gain and attention are two key neural processes that mediate the cue validity effect on performance in Posner-like tasks. In short, a perceptual expectation induced by a cue increases the response gain of neural populations called prediction units, generating a prediction signal. This prediction signal is conveyed to lower-level prediction-error units, which encode the contextual relevance and generate a prediction-error signal. Input of highly reliable prediction-error signals to higher-level prediction units boost attention and the drive to revise perceptual expectations.

Thus, recurrent messaging between prediction and prediction-error units adaptively adjusts perceptual expectations to contextual demands by optimizing processing gain and attentional responses to incoming sensory input. Simulated electrophysiological responses of the Posner paradigm align modulation of *prediction units* with increased amplitudes following validly relative to invalidly cued targets and higher processing gain. Subsequent modulation of *prediction-error units* is aligned with increased amplitudes for invalidly relative to validly cued targets and increases in attention^12^. Several results of experimental studies support these simulation findings. That is, increased anterior negativity in the N1 window (N1a) has been reported following validly relative to invalidly predicted targets^15^ and for targets that are more frequently presented than other targets^16,17^. The N1a subcomponent peaks between 100 and 200 ms post-stimulus over anterior electrodes and forms the visual N1 wave together with at least two other distinct subcomponents^18^. In Posner-like tasks, increases in N1a amplitude in the valid compared to the invalid condition may thus index increases in processing gain. The N1a component is followed in time by an increase in posterior contralateral negativity (N1pc)^18^. Increased N1pc amplitudes have been observed for invalidly relative to validly cued targets^19^ and would reflect increases in stimulus-driven selective attention^20^. Thus, this suggests that in Posner-like tasks increases in N1pc amplitude in the invalid as compared to the valid condition can be interpreted as a neural correlate of stimulus-driven attention. To summarize, two neural processes which contribute to faster performance on Posner-like tasks are: (1) processing gain, indexed by an increased N1a amplitude in the valid compared to the invalid condition, and (2) increased attention indexed by an increased N1pc amplitude in the invalid compared to the valid condition.

How do processing gain and attention subsequently influence behavioral responses on Posner-like tasks? Pre-existing perceptual expectations bias sensory processing before the onset of subsequent perceptual decision-making^21^. On the neural level, pre-existing perceptual expectations prioritize processing of expected sensory signals by increasing processing gain of prediction units, which generates a prediction signal^12^. This prediction signal influences the time-course of perceptual decision making through the so-called perceptual decision bias towards a response^22^. A perceptual decision bias is defined as a starting point of perceptual decision making, from which sensory evidence starts to accumulate towards a response threshold^23^. An increased perceptual decision bias will thus bias sensory evidence towards a response threshold. The perceptual decision bias towards a response and other parameters that influence the time-course of perceptual decision making can be estimated with Diffusion Drift Models (DDM) based on reaction time and performance accuracy data. The most important DDM parameters are: (1) drift rate *v*, i.e. the rate at which evidence for a response option accumulates: (2) threshold separation *a*, i.e. the amount of input needed to activate a response, and (3) the starting point parameter *z*_*r*_, which reflects the perceptual decision bias. Among these parameters, the perceptual decision bias most strongly accounts for the *cue validity effect* on visuospatial orienting task performance^13^, with decreases in the perceptual decision bias covarying with decreases in the probability of a valid cue, and a reduced *cue validity* effect on reaction time performance^14^. Less precise perceptual expectations in ASD may therefore lead to slower visuospatial orienting on a behavioral level^3^, because reduced processing gain results in suboptimal perceptual decision bias.

One previous ERP study investigated visuospatial orienting in ASD with a Posner-like task. Findings indeed suggest impairments in processing gain and perceptual decision making during visuospatial orienting in ASD^24^. That is, Lateralized Readiness Potential (LRP) amplitudes following invalidly cued targets were increased in ASD relative to neurotypical participants (NT). Increased LRP amplitudes correlate with lower perceptual decision bias^25^ and indicate a prolonged duration of perceptual decision making^26^. In addition, increased anterior N1 (N1a) amplitudes following invalidly cued targets were observed in ASD compared to NT^24^. This suggests an increased processing of invalidly cued targets in ASD and thus a reduced gain in processing validly relative to invalidly cued targets. In sum, results of this single EEG study on a Posner-like task suggest that reduced processing gain may attenuate the perceptual decision bias across invalid trials and explain slower visuospatial orienting in ASD. However, electrophysiological indices of attention, such as the N1pc, were not examined in this study.

During invalid trials on a Posner-like task, reliably perceived discrepancies between incoming sensory input and prior perceptual expectations boost attention. This activates the drive to update perceptual expectations and adapt motor behavior, which eventually attenuates attentional responses to invalidly cued targets^12,27^. Perceptual expectations are updated at reduced rates in individuals with ASD^6,7^. On Posner-like tasks, this implies that for individuals with ASD invalidly cued targets may remain relatively more “surprising” for longer time periods. Regarding attention on Posner-like tasks, this predicts an overall increased attention to invalidly relative to validly cued targets in ASD relative to NT.

To summarize, reduced perceptual expectations in ASD may differentially influence at least two neural processes during visuospatial orienting performance relative to NT. First, reduced processing gain may lead to suboptimal perceptual decision bias and slower behavioral performance. Second, overall increases in attention may accelerate performance and partly compensate slower behavioral performance. So far, one previous study examined the cue validity effect on processing gain in ASD^24^, but did not examine electrophysiological indices of attention, such as the N1pc, and the interrelation with the perceptual decision bias. To address these gaps in the existing literature and empirically test the outlined model above, the present study compared the cue validity effect on processing gain, attention and the perceptual decision bias between children and adolescents with ASD (N = 23) and neurotypical children and adolescents (NT) (N = 23). Across groups, we hypothesized an increased processing gain, a higher perceptual bias and shorter behavioural responses for validly relative to invalidly cued targets, and increased attention for invalidly relative to validly cued targets. We predicted increased N1a amplitudes, starting values and shorter mean RT in the valid as compared to the invalid condition, and increased N1pc amplitudes in the invalid as compared to the valid condition. Second, we hypothesized that the cue validity effect on visuospatial orienting performance in ASD is explained by both a reduced processing gain and compensatory increases in attention. In ASD relative to NT, we therefore predicted a reduced increase in the N1a amplitude in the valid as compared to the invalid condition, a larger increase in the N1pc amplitude in the invalid as compared to the valid condition, and an even slower mean RT and reduced perceptual decision bias in the invalid as compared to the valid condition. Third, to exploratorily investigate how behavioural responses are influenced by the effect of perceptual expectations on processing gain and by attention, we examined whether processing gain and attention differentially correlated with perceptual decision biases and mean RT across and within groups. We expected that increases in processing (N1a amplitude) were correlated with a higher perceptual decision bias and faster mean RT and increases in attention (N1pc amplitude) with faster mean RT across groups.

## Methods

### Participants

The study sample consisted of 23 children and adolescents with ASD and 23 neurotypical children and adolescents (NT) (see Table 1). Participants were recruited from the Department of Child and Adolescent Psychiatry, Psychosomatics and Psychotherapy, University Hospital Frankfurt, Goethe-University, Frankfurt, Germany. The research project was approved by the local ethics committee of the Medical Faculty at the University Clinic Frankfurt (reference number 416/17) and carried out in accordance with the Declaration of Helsinki (World Medical Association, 2013). Written informed consent for participation and publication and was obtained from all parents or legal guardians. Inclusion criteria for both groups were: Intelligence Quotient (IQ) ≥ 70, age between 10 and 17 years and 11 months, and normal or corrected-to-normal vision. Exclusion criteria for both groups were any neurological conditions, birth weight ≤ 2000 g, born before the 32nd week of pregnancy, psychotropic medication, history of epilepsy, or a first degree relative with history of epilepsy. Inclusion criteria for the ASD group was an expert ICD-10 diagnosis of autism, atypical autism/PDD-NOS or Asperger syndrome based on the Autism Diagnostic Observation Schedule, second version (ADOS-2)^28^ and the Autism Diagnostic Interview-Revised (ADI-R)^29^. Exclusion criteria for the ASD group were schizophrenia, bipolar disorder, current depressive episodes, anxiety disorders, obsessive compulsive disorder, conduct disorder and addiction disorders. Ten participants additionally met DSM-5 Attention-Deficit/ Hyperactivity Disorder (ADHD) diagnostic criteria (combined type ADHD: *N* = 3; predominantly inattentive ADHD: *N* = 5; predominantly hyperactive/impulsive ADHD-H/I: *N* = 2). NT participants were excluded if parent ratings of one of the eight Child Behaviour Checklist (CBCL) syndrome scales exceeded the clinical cut-off (T-score > 70)^30^.

**Table 1:** Study sample characteristics.

	ASD (*n* = 23)		NT (*n* = 23)		Test statistic	*p*	Group difference
	*M*	*SD*	*M*	*SD*			
Age	15.26	2.21	15.55	1.59	*K-W* χ^2^ (1,* N* = 46) = 0.01	0.930	–
IQ	96.28	13.70	105.98	10.13	*F*(1,44) = 7.45	0.009	ASD < NT
CBCL total t-score	65.48	9.09	42.87	7.83	*F*(1,44) = 81.69	< 0.001	ASD > NT
SRS-16 total raw score	36.22	10.94	16.05	5.84	*K-W* χ^2^ (1, *N* = 45) = 29.05	< 0.001	ASD > NT
Inattention symptom severity (FBB-ADHD)	12.65	5.87	2.70	3.01	*K-W* χ^2^ (1, *N* = 46) = 6.34	< 0.001	ASD > NT
Hyperactivity/impulsivity symptom severity (FBB-ADHD)	8.78	6.57	0.57	1.46	*K-W* χ^2^ (1, *N* = 46) = 27.23	< 0.001	ASD > NT
**Sex**	*n*		*n*		χ^2^ (1, *N* = 46) = 0.169	0.681	–
Female	3		4				
Male	20		19				

ASD: Autism Spectrum Disorder; NT: neurotypical control group; IQ: intelligence quotient; K-W χ^2^, CBCL: Child Behaviour Checklist/4-18R: SRS-16: Social Responsiveness Scale - short form; ADHD: Attention-Deficit/Hyperactivity Disorder; FBB-ADHD: FBB-ADHD rating scale for parents.

### Materials and design

#### Phenotypic data

ADHD symptom severity scores were obtained by the ADHD rating scale for parents (FBB-ADHD)^31^. Parents rated ADHD symptom severity during the past 6 months on 18 items based on DSM-IV and ICD-10 criteria, scaled from 0 (non-existent) to 3 (strongly pronounced). Social communication symptom severity scores were obtained using the short version of the Social Responsiveness Scale-short form (SRS-16)^32^. Parents rated social communication symptom severity during the past six months on 16 items, scaled from 0 (never true) to 3 (almost always true). For one participant (NT) the SRS-16 was missing. Group differences in SRS were calculated without this participant (see Table 1). Impairments in behavior, emotion and somatic functioning during the past 6 months were obtained with the Child Behaviour Checklist/4-18R (CBCL/4-18R)^30^. Parents rated 99 items, scaled from 0 (not true) to 2 (very true or often true).

#### IQ

IQ was estimated using the two subscales Vocabulary and Picture Completion of the Wechsler Intelligence Scale for Children (3rd ed.)^33^.

#### Procedure and experimental paradigm

Participants completed a visuospatial orienting task in a quiet testing room as part of a larger test battery. For a previous publication on a different task see^34^.

**Figure 1:**
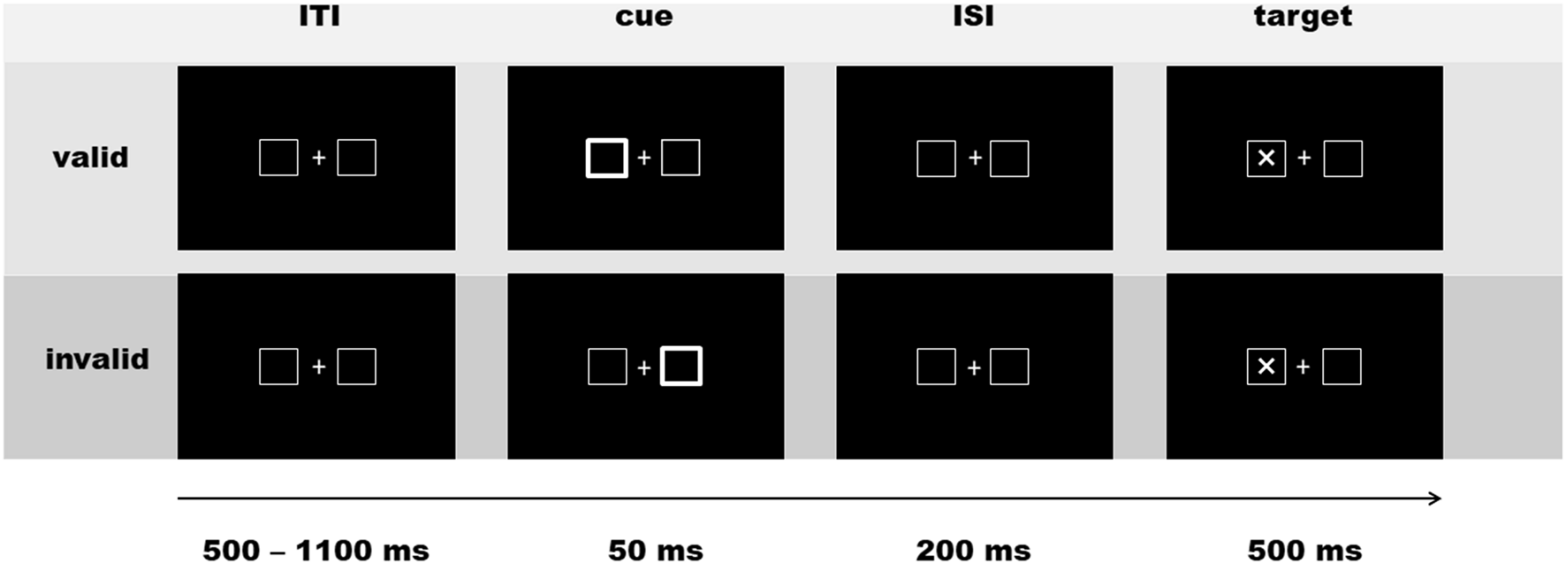
Experimental paradigm. ITI: inter-trial-interval; ISI: inter-stimulus-interval; ms: millisecond.

EEG, reaction time and accuracy performance were recorded for all participants while performing a task based on the Posner cueing paradigm^35^ (see Fig. 1). First, a neutral screen with a fixation cross was shown with two boxes presented to the left and right of a fixation cross. After an inter-trial-interval (ITI) varying between 500 and 1100 ms, a cue frame surrounding one of the two boxes predicted an upcoming target location in about 70% of the trials (valid condition; n = 178). In the other 30% of the trials, this cue incorrectly predicted the upcoming target location (invalid condition; n = 78). Cue stimuli were presented for 50 ms, target stimuli (an “X” appearing inside one of the boxes) for 500 ms, separated by a 200 ms inter-stimulus interval, making a cue-to-target stimulus onset asynchrony (SOA) of 250 ms. Participants were instructed to fixate their eyes on a central cross and respond to target stimuli by pressing a button with the hand that corresponded to target location (i.e. left or right). Participants additionally completed the same number of trials with a shorter SOA of 100 ms. Trials with a different SOA were presented in randomized order. Only trials with a SOA of 250 ms were analyzed in the present study, to avoid superposition of target-locked EEG components of interest on electrophysiological activity evoked by the preceding cue. The task was divided into three blocks that lasted approximately 5 min each with short breaks in between.

#### EEG recording

EEG data were recorded with 64 sintered Ag/AgCl electrodes embedded in an elastic cap according to the extended International 10–20 system with FCz as recording-reference. One separate electrode was placed approximately 1 cm below the right eye to record blinks. Impedances were kept below 10 kΩ. Signal was amplified and digitized using Brain Vision BrainAmp MR-Plus amplifiers and Brain Vision Recorder software, with an online anti-aliasing band pass filter (low cut-off: 0.01 Hz; high cut-off: 250 Hz; slope: 12 dB/octave) and a sampling rate of 1000 Hz.

#### EEG data pre-processing

Offline processing of the EEG data was performed using BrainVision Analyzer version 2.2. Data were re-referenced offline to the average of the left and right mastoid electrodes (TP9; TP10) and down-sampled to 500 Hz. After applying a high-pass filter of 0.1 Hz (8th order Butterworth zero phase-shift filter), gross artefacts such as extensive muscle contractions and movement artefacts were manually removed by visual inspection. Subsequently, independent component analysis (ICA) (infomax algorithm) was applied to identify and subsequently remove artifacts consisting of eye blinks and eye movements, muscle tension, and other artefacts such as line noise. Data for correct responses were segmented into epochs of 1470 ms (200 ms pre-cue to 1270 ms post-cue onset) separately for condition (valid; invalid) and target location (left; right), baseline-corrected using the mean of the 200 ms interval before cue onset, and low-pass filtered with 30 Hz (8th order Butterworth zero phase-shift filter). A semi-automatic artefact rejection on the resulting segments was applied using the following criteria: voltage steps over 50 µV/ms, datapoints with amplitudes higher than 200 µV or lower than − 200 µV and a minimum activity difference of 0.5 µV per 100 ms. Finally, segments were manually inspected for remaining artifacts. Segments with artefacts such as eye movements or blinks in the time period between cue onset and the end of target presentation were rejected. Participants for whom more than one third of the segments were rejected were excluded from further data analysis (NT; *N* = 1).

#### N1a

The N1a component was characterised by an anterior distribution (see Supplementary Figs. S1–S3 online), in line with previous results on visuospatial orienting in ASD^24^. The average signal was calculated for a cluster of left hemisphere frontal electrodes (F1; F3; AF3), a cluster of right hemisphere frontal electrodes (F2; F4; AF4), and for the midline electrode Fz. N1a mean amplitudes were calculated with the mean adaptive mean amplitude method^36^. Individual peak latencies in each condition were manually detected within participants and a pre-specified time window between 80 and 180 ms^24^. Based on the grand average (GA) waveforms across groups (see Fig. S1 online), N1a mean amplitudes were extracted as the mean signed area under the curve ± 12 ms around the individual peak latencies. N1a onset latency was estimated as the time point when 50% of the amplitude had been developed^37^.

#### Event-related-lateralizations (ERL): N1pc

The N1pc was measured as the contralateral minus ipsilateral difference wave [for grand average (GA) waveform and scalp topographies see Supplementary Figs. S4–S6 online] between two electrode clusters of three lateral posterior electrodes (left PO7/P7/P5 and right PO8/P8/P6)^16^. ERL was calculated by subtracting the signal contralateral to the target from the ipsilateral signal for both target locations separately and then averaging these differences:

$$\mathbf{E}\mathbf{R}\mathbf{L} =\frac{(\mathrm{P}7/\mathrm{PO}7/\mathrm{P}5 -\mathrm{P}8/\mathrm{PO}8/\mathrm{P}6 Right Target) +(\mathrm{P}8/\mathrm{PO}8/\mathrm{P}6 -\mathrm{P}7/\mathrm{PO}7/\mathrm{P}6 Left Target)}{2}$$

N1pc mean amplitudes were calculated with the adaptive mean amplitude method^36^. Individual peak latencies in each condition were manually detected in ERL average waveforms within participants and a pre-specified time window of 120–220 ms. N1pc mean amplitudes were extracted as the mean signed area under the curve ± 12 ms around the individual peak latencies for each participant in each condition. As no previous study examined the N1pc in a sample of children and adolescents, time windows were based on the GA waveforms in the valid condition and the invalid condition across groups (see Supplementary Fig. S4 online). Onset latencies of all ERL components were estimated with 50% peak latencies^37^.

#### Estimation of decision bias and drift rates

Diffusion drift models estimate the course of perceptual decision making from reaction time and accuracy performance data^23^. Parameters describing this diffusion process are: *drift rate (v),* which is rate at which sensory evidence accumulates; threshold separation (*a*), which defines the amount of input needed to activate a response; and *starting point* (*z*_*r*_), the decision bias. In addition, a non-decisional constant (t_0_) estimates the duration of processes related to perception, attention and motor preparation^38^. Model parameters were estimated using the Fast-dm modelling technique^39^. The fitted model was based on a previously published model using a similar task (see^13^) using the Kolmogorov–Smirnov (KS) optimization criterion. Correct responses were defined as the upper threshold and incorrect response as the lower threshold. In line with a previous study^13^, the effect of perceptual expectation on decisional bias (starting point: *z*_*r*_) and the rate of evidence accumulation (drift rate: *v*) were estimated separately for each condition. Non-decisional constant (t0), threshold separation *(a)* and inter-trial variability of non-decisional constant (*s*_*t0*_) were estimated across conditions. For each participant model fit was evaluated with a statistical threshold obtained with Monte Carlo Simulations^38,40^ using the construct-sample tool of fast-dm^39^ (see Supplementary Information online for more details).

#### Statistical analysis

Participants with less than 70% accurate responses (ASD: *N* = 2; NT: *N* = 1) and trials with responses shorter than 200 ms (6.8%) and longer than 1000 ms (0.8%) were removed from the analysis to eliminate guesses^40^. Main effects and group differences in the effect of cue validity on behavioral performance (mean reaction time (RT); percentage correct responses), EEG measures (N1a; N1pc), and diffusion drift parameters (starting values; drift rates) were analysed with repeated measures ANCOVA using the afex package^41^ in R 4.0.3^42^. The ANCOVA models to analyse the behavioral performance and DDM parameters included condition as a within-subject factor (valid; invalid) and group as a between-subjects factor (ASD; NT). In line with previous literature, electrode location (contralateral; midline; ipsilateral) was included as a within-subject factor for the analysis of the cue validity effect on the N1a amplitude^17^. To control for the effect of ADHD symptom severity on reaction time, accuracy performance^43^ and EEG components^44–46^, a combined measure of ADHD symptom severity (i.e. inattentive and hyperactive/impulsive symptoms) was included as a between-subjects factor, for main effect and interactions. In addition, all models included age as co-variate of no-interest. For all ANCOVA models, Greenhouse–Geisser corrected *p*-values were reported. Generalized eta squared (η^2^G) and partial eta squared (η_p_^2^) were calculated as measures of effect size using the afex package^41^ in R 4.0.3^42^. Post hoc tests were calculated using package emmeans^47^. A priori sensitivity power analyses were performed with G*Power 3^48^. Planned analyses had a sensitivity to detect small statistical effects of cue validity (Cohen’s f^2^ = 0.16) and group differences in cue validity (Cohen’s f^2^ = 0.19). P-values of all main and interaction effects of interest and pairwise comparisons were corrected using the false discovery rate method^49^, separately for the behavioral (n of comparisons = 8) and EEG measures (n of comparisons = 8).

To exploratory examine whether processing gain and attentional allocation differentially influenced behavioral performance, correlations between N1a amplitude and N1pc amplitude with starting values and mean RT were calculated across and within groups. Correlations were estimated as standardized regression coefficients using linear mixed regression models with the R package lme4^50^ and participant ID as random intercept. Standardized regression coefficients across and within groups were estimated with separate models. In each model, N1a or N1pc amplitude was the dependent variable, with either starting value or mean RT as continuous predictor, condition (valid; invalid) as a categorical predictor, and age and ADHD symptom severity as continuous co-variates of no-interest. Models across groups included an interaction term for between-subject factor group (ASD; NT) and condition (valid; invalid) to control for variation due to group differences in the effect of perceptual expectation on N1a and N1pc amplitude, respectively. N1a amplitude models included electrode location (contralateral; midline; ipsilateral) as a within-subject factor. All continuous variables were z-standardized. Standardized regression coefficients and 95% Confidence Intervals (95% CI) were calculated within and across groups. 95% CI within groups were used to assess group differences in the correlations.

Finally, three participants in the ASD group were on stimulant medication during the task, as the burden to temporarily withdraw from stimulant treatment was too high. To estimate whether acute effects of stimulant use in the ASD group influenced the main outcome EEG measures^51^, all analyses regarding the N1a and N1pc amplitude were recomputed without those participants (*N* = 3).

## Results

Sample characteristics are reported in Table 1. Groups did not differ in age and sex ratio. IQ scores were lower in ASD compared to NT. ASD scored higher than NT on all clinical symptom measures. Mean reaction time (mean RT) did not differ between groups (Table 2).

**Table 2:** The cue validity effect (valid cue vs. invalid cue) and group differences in the cue validity effect on mean reaction time.

Effect	*F*(1,41)	*p*	η_p_^2^	η^2^G
**Main effects—between subjects**				
Group	0.40	0.533	0.010	0.007
ADHD symptom severity	1.67	0.204	0.039	0.030
Age	8.10	0.007	0.144	0.165
**Main effects—within subjects**				
Cue validity	33.54	< 0.001	0.450	0.055
**Interaction effects**				
Group × cue validity	0.43	0.589	0.010	< 0.001
Group × ADHD symptom severity	0.43	0.516	0.010	0.008
Cue validity × ADHD symptom severity	2.04	0.161	0.047	0.004
Group × cue validity × ADHD symptom severity	0.07	0.792	0.002	< 0.001

ADHD: Attention-Deficit/Hyperactivity Disorder.

### The cue validity effect across groups

#### The cue validity effect on task performance: mean RT and performance accuracy

All results on the effect of perceptual expectations on mean RT and accuracy are displayed in Tables 2 and 3, respectively.

**Table 3:** The cue validity effect (valid cue vs. invalid cue) and group differences in the cue validity effect on performance accuracy.

Effect	*F*(1,41)	*p*	η_p_^2^	η^2^G
**Main effect—between subjects**				
Group	0.57	0.454	0.014	0.009
ADHD symptom severity	2.28	0.129	0.053	0.036
Age	1.87	0.179	0.044	0.030
**Main effect—within subjects**				
Cue validity	26.18	< 0.001	0.390	0.137
**Interaction effects**				
Group × cue validity	0.64	0.588	0.015	0.004
Group × ADHD symptom severity	0.14	0.713	0.003	0.002
Cue validity × ADHD symptom severity	1.79	0.189	0.042	0.011
Group × cue validity × ADHD symptom severity	1.47	0.233	0.035	0.009

ADHD: Attention-Deficit/Hyperactivity Disorder.

Results showed a main effect of cue validity on mean RT (η_p_^2^ = 0.450, η^2^G = 0.055; see Table 2). Mean RT was faster following validly compared to invalidly cued targets, *mean diff* = − 50.4 ms (ms), 95% CI [− 68, − 32.9], *p* < 0.001. Results showed a main effect of cue validity on performance accuracy (η_p_^2^ = 0.390, η^2^G = 0.137; see Table 3). Participants responded more accurately to validly compared to invalidly cued targets, *mean diff* = 8.8% (percent correct), 95% CI [5.33, 12.30], *p* < 0.001.

#### The effect of cue validity on perceptual decision making: starting points and drift rate

DDM model fit was significant for all participants (see Supplementary information online for more details). Results on the effect of cue validity on starting point estimates and drift rate are displayed in Tables 4 and 5, respectively. For a descriptive summary of all DDM parameters see Table 6.

**Table 4:** The cue validity effect (valid cue vs. invalid cue) and group differences in the cue validity effect on decisional bias (z_r_).

Effect	*F*(1,41)	*p*	η_p_^2^	η^2^G
**Main effect—between subjects**				
Group	0.41	0.532	0.10	0.007
ADHD symptom severity	0.00	0.999	< 0.001	< 0.001
Age	1.26	0.269	0.030	0.021
**Main effect—within subjects**				
Cue validity	23.96	< 0.00	0.369	0.143
**Interaction effects**				
Group × cue validity	0.18	0.671	0.004	0.001
Group × ADHD symptom severity	0.45	0.505	0.011	0.007
Cue validity × ADHD symptom severity	0.01	0.937	< 0.001	< 0.001
Group × cue validity × ADHD symptom severity	0.79	0.379	< 0.001	< 0.001

ADHD: Attention-Deficit/Hyperactivity Disorder.

**Table 5:** The cue validity effect (valid cue vs. invalid cue) and group differences in the cue validity effect on drift rate (v).

Effect	*F*(1,41)	*p*	η_p_^2^	η^2^G
**Main effect—between subjects**				
Group	2.06	0.159	0.048	0.024
ADHD symptom severity	2.89	0.097	0.066	0.033
Age	4.29	0.045	0.095	0.049
**Main effect—within subjects**				
Cue validity	3.28	0.154	0.074	0.028
**Interaction effects**				
Group × cue validity	0.60	0.588	0.015	0.005
Group × ADHD symptom severity	1.64	0.208	0.038	0.019
Cue validity × ADHD symptom severity	1.32	0.258	0.031	0.012
Group × cue validity × ADHD symptom severity	1.82	0.185	0.042	0.016

ADHD: Attention-Deficit/Hyperactivity Disorder.

**Table 6:** Descriptive summary of estimated Diffusion Drift Model parameters in ASD and NT.

DDM parameter	ASD (*n* = 23)		NT (*n* = 23)	
	*M*	*SD*	*M*	*SD*
**Drift rate (*v*)**				
Valid	4.47	1.65	4.41	0.88
Invalid	5.86	1.78	6.08	1.38
**Starting point (*z***_***r***_**)**				
Valid	0.71	0.10	0.74	0.11
Invalid	0.42	0.16	0.49	0.18
Threshold separation (*a*)	1.36	0.27	1.60	0.47
Non-decisional constant (*t0*)	0.27	0.03	0.27	0.02
Inter-trial-variability non-decisional constant (*s*_*t0*_)	0.12	0.07	0.09	0.03

DDM: diffusion drift model; ASD: Autism Spectrum Disorder; NT: neurotypical.

A main effect of cue validity on starting points was found (η_p_^2^ = 0.369, η^2^G = 0.143; see Table 4). Starting points were higher following validly compared to invalidly cued targets, *mean diff* = 0.23, 95%, CI [0.14, 0.33], *p* < 0.001. In contrast, the effect of cue validity on drift rates was not significant (η_p_^2^ = 0.074; η^2^G = 0.028; see Table 5).

#### The cue validity effect on processing gain: N1a amplitude

Results on the cue validity effect on N1a amplitude are displayed in Table 7. Results of the analysis without participants with ASD on stimulant medication are displayed in Table S1.

**Table 7:** Cue validity effect and group differences in the cue validity effect on N1a amplitude.

Effect	*F* (1,41)	*p*	η_p_^2^	η^2^G
**Main effects—between subjects**				
Group	0.08	0.783	0.002	0.001
ADHD symptom severity	0.21	0.652	0.005	0.003
Age	8.59	0.006	0.173	0.137
**Main effects—within subjects**				
Cue validity	5.55	0.037	0.119	0.016
	***F* (1.64, 67.34)**			
Electrode location	3.47	0.045	0.078	0.002
**Interaction effects**	***F* (1,41)**			
Group × cue validity	4.88	0.044	0.106	0.014
Group × ADHD symptom severity	0.33	0.569	0.008	0.005
Cue validity × ADHD symptom severity	3.81	0.058	0.085	0.011
Cue validity × group × ADHD symptom severity	4.97	0.031	0.108	0.014
	***F* (1.64, 67.34)**			
Group × electrode location	2.04	0.146	0.047	0.001
ADHD symptom severity × electrode location	1.26	0.285	0.30	< 0.001
Group × ADHD symptom severity × electrode location	0.07	0.906	0.002	< .001
	***F* (1.74, 71.40)**			
Electrode location × cue validity	1.35	0.265	0.032	< .001
Group × cue validity × electrode location	4.77	0.015	0.104	0.001
ADHD symptom severity × cue validity × electrode location	7.66	0.002	0.157	0.002
Group × cue validity × electrode location × ADHD symptom severity	1.52	0.227	0.036	< 0.001

ADHD: Attention-Deficit/Hyperactivity Disorder.

Results showed a main effect of cue validity on N1a amplitude (η_p_^2^ = 0.119; η^2^G = 0.016; see Table 7). N1a amplitudes were increased after validly predicted targets relative to invalidly predicted targets, *mean diff* = − 1.36 μV, 95% CI [− 0.19, − 2.53], *p* = 0.037. Results did not change when participants on stimulant medication were excluded from the analysis, *mean diff* = − 1.20 μV, 95% CI [− 0.11, − 2.30], *p* = 0.023. There was no main effect of cue validity on N1a latency onset (see Supplementary Table S2 online).

#### The cue validity effect on attentional allocation: N1pc amplitude

Results on the cue validity effect on N1pc amplitude are reported in Table 8. Results of the analysis without participants with ASD on stimulant medication are displayed in Table S3.

**Table 8:** Cue validity effect and group differences in the cue validity effect on N1pc amplitude.

Effect	*F* (1,41)	*p*	η_p_^2^	η^2^G
**Main effects—between subjects**				
Group	0.10	0.749	0.003	0.002
ADHD symptom severity				
Age	1.22	0.275	0.028	0.022
**Main effect—within subjects**				
Cue validity	16.30	< 0.001	0.285	0.071
**Interaction effects**				
Group × cue validity	5.70	0.037	0.122	0.026
Group × ADHD symptom severity	0.06	0.807	0.001	0.001
ADHD symptom severity × cue validity	2.32	0.136	0.053	0.011
Group × cue validity × ADHD symptom severity	1.54	0.222	0.036	0.007

ADHD: Attention-Deficit/Hyperactivity Disorder.

There was a main effect of cue validity on N1pc amplitude (η_p_^2^ = 0.285; η^2^G = 0.071; Table 8). N1pc amplitude was increased following invalidly cued targets as compared to validly cued targets, *mean diff* = − 3.46, 95% CI [− 5.19, − 1.73], *p* < 0.001. Results did not change when participant on stimulant medication were excluded from the analysis, *mean diff* = − 3.62, 95% CI [− 5.11, − 2.12], *p* < 0.001. Results showed no significant effect of cue validity on N1pc onset latency (see Supplementary Table S4 online).

#### Correlations between N1a and N1pc amplitude with starting point estimates and mean RT

Across groups, starting values were not correlated with N1a amplitudes, b* = − 0.05, *SE* = 0.07, 95% CI [− 0.19, 0.08], and N1pc amplitudes, b* = − 0.05, *SE* = 0.12, 95% CI [− 0.28, 0.18]. Results did not change when participants on stimulant medication were excluded from the analysis, N1a amplitude: b* = − 0.01, *SE* = 0.07, 95% CI [− 0.16, 0.13]; N1pc amplitude: b* = − 0.15, *SE* = 0.12, 95% CI [− 0.37, 0.10]. Mean RT furthermore did not correlate with N1a amplitudes, b* = 0.16, *SE* = 0.10, 95% CI [− 0.02, 0.35]. When participants on stimulant medication were excluded from the analysis, however, faster mean RT correlated with increased N1a amplitudes, b* = 0.20, *SE* = 0.10, 95% CI [0.01, 0.41]. Faster mean RT additionally correlated with more increased N1pc amplitudes, b* = 0.35, *SE* = 0.12, 95% CI [0.11, 0.58]. Results did not change when participants on stimulant medication were excluded from the analysis, b* = 0.34, *SE* = 0.14, 95% CI [0.09, 0.60].

### Group differences in the cue validity effect on task performance, perceptual decision making, processing gain and attentional allocation

#### Group differences in the cue validity effect on task performance: mean RT and performance accuracy

Results on the cue validity effect on mean RT and accuracy are displayed in Tables 2 and 3, respectively. No group differences were found for the cue validity effect on mean RT (see Table 2) and accuracy (see Table 3).

#### Group differences in the cue validity effect on perceptual decision making: starting points and drift rate

Results on the cue validity effect on starting point estimates and drift rate are displayed in Tables 4 and 5, respectively. No group differences were found in the cue validity effect on starting points (see Table 4) and drift rate (see Table 5).

#### Group differences in the cue validity effect on processing gain: N1a amplitude

Results on the cue validity effect on N1a amplitudes are displayed in Table 7. Results of the analysis without participants with ASD on stimulant medication are displayed in Table S1.

**Figure 2:**
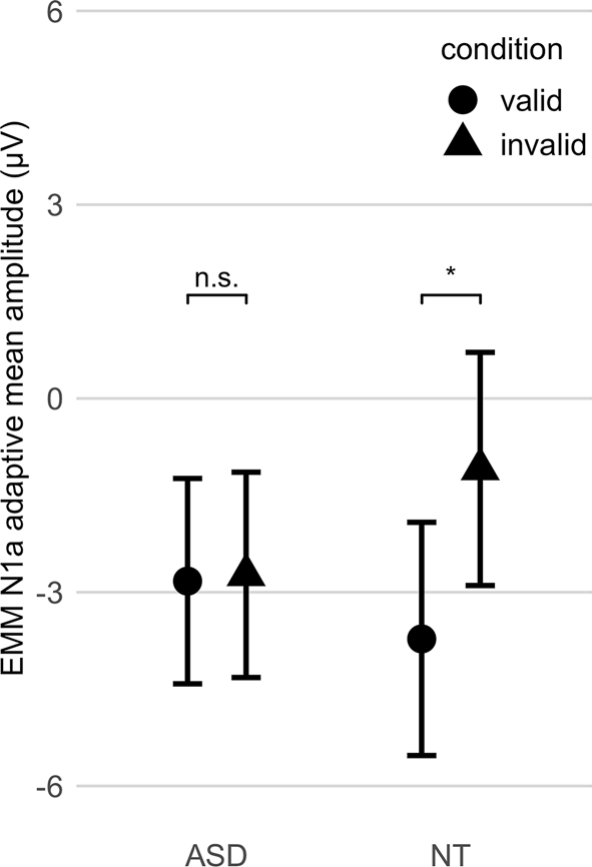
Significant modulation of N1a amplitude by cue validity in NT, but not in ASD. EMM: estimated marginal means; * p < 0.05; ns: not significant; ASD: Autism Spectrum Disorder; NT: neurotypical children; error bars: 95% confidence intervals. This figure was made using the R package ggplot2^68^.

The cue validity effect on N1a differed between groups (η_p_^2^ = 0.106; η^2^G = 0.014; see Table 7 and Fig. 2). In NT, N1a amplitudes were increased following validly cued compared to invalidly cued targets, *mean diff* = − 2.63 μV, 95% CI [− 0.56, − 4.70], *p* = 0.037. In ASD, no difference was observed between N1a amplitudes following validly relative to invalidly cued targets, *mean diff* = − 0.097 μV, 95% CI [− 1.16, 0.10], *p* = 0.854. Results did not change when participants with stimulant medication were excluded from the analysis, *mean diff* = − 0.133 μV, 95% CI [− 1.16, 1.33], *p* = 0.822. Results showed no group differences in the cue validity effect on N1a latency onset (Table S2).

#### Group differences in the cue validity effect on attentional allocation: N1pc amplitude

Results on the cue validity effect on N1pc amplitudes are displayed in Table 8. Results of the analysis without participants with ASD on stimulant medication are displayed in Table S3.

**Figure 3:**
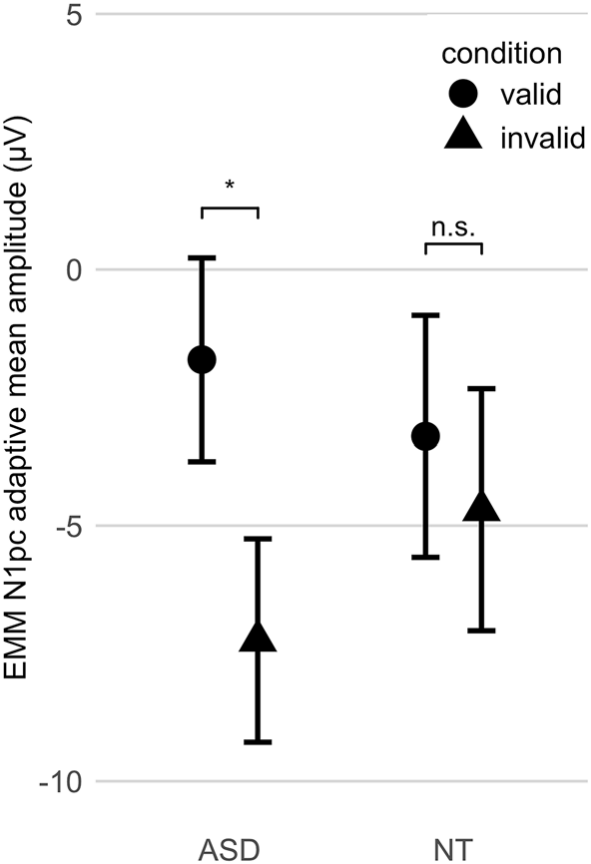
Significant modulation of N1pc amplitude by cue validity in ASD, but not in NT. EMM: estimated marginal means; * *p* < 0.001; n.s.: not significant; ASD: Autism Spectrum Disorder; NT: typically developing children; error bars: 95% confidence intervals. This figure was made using the R package ggplot2^68^.

The cue validity effect on N1pc differed between groups (η_p_^2^ = 0.122; η^2^G = 0.026; Table 8; Fig. 3). In NT, no significant difference in N1pc amplitude was found between invalidly and validly cued targets, *mean diff* = − 1.44 μV, 95% CI [− 4.50, 1.63], *p* = 0.399. In ASD, N1pc amplitudes were increased following invalidly relative to validly cued targets, *mean diff* = − 5.49 μV, 95% CI [− 7.06, − 3.91], *p* < 0.001. Results did not change when participants with stimulant medication were excluded from the analysis, *mean diff* = − 5.43 μV, 95% CI [− 7.06, 3.80], *p* < 0.001. Results showed no significant group differences in the cue validity effect on N1pc onset latency (see Table S4).

#### Group differences in correlations between N1a and N1pc amplitudes with starting values and mean RT

Correlations between N1a amplitude with starting values differed between groups based on the 95% CI. In NT, more negative N1a amplitudes correlated with higher starting values, b* = − 0.22, *SE* = 0.10, 95% CI [− 0.42, − 0.02]. In ASD, N1a amplitudes did not correlate with starting values, b* = 0.09, *SE* = 0.10, 95% CI [− 0.08, 0.28]. When participants on stimulant medication were excluded, increased N1a amplitudes in ASD correlated with higher starting values, b* = 0.22, *SE* = 0.11, 95% CI [0.43, 0.02]. N1pc amplitudes did not correlate with starting values in NT, b* = − 0.08, *SE* = 0.18, 95% CI [− 0.41, 0.25], and in ASD, b* = − 0.03, *SE* = 0.17, 95% CI [− 0.34, 0.30]. Results did not change when participants on stimulant medication were excluded from the analysis, b* = − 0.23, *SE* = 0.17, 95% CI [− 0.54, 0.14]. Furthermore, correlations between N1a amplitude with mean RT additionally differed between groups. In NT faster mean RT correlated with increased N1a amplitudes, b* = 0.65, *SE* = 0.14, 95% CI [0.38, 0.92]. In ASD, mean RT was not correlated with N1a amplitudes, b* = − 0.10, *SE* = 0.14, 95% CI [− 0.35, 0.19]. Results did not change when participants on stimulant medication were excluded from the analysis, b* = − 0.06, *SE* = 0.15, 95% CI [− 0.35, 0.27]. Finally, correlations between mean RT and N1pc were not different between groups. In ASD, slower mean RT correlated with more positive N1pc amplitudes, b* = 0.44, *SE* = 0.20, 95% CI [0.06, 0.82]. In NT, mean RT was not significantly correlated with N1pc amplitudes, b* = 0.23, *SE* = 0.15, 95% CI [− 0.05, 0.51]. When participants on stimulant medication were excluded from the analysis, correlations between mean RT and N1pc amplitudes in ASD were similar in magnitude, but not significantly different from zero, b* = 0.44, *SE* = 0.24, 95% CI [− 0.01, 0.89].

## Discussion

The present study examined the effect of perceptual expectations on processing gain (N1a), attention (N1pc) and the perceptual decision bias (starting values) during visuospatial orienting in ASD and NT. Across groups, results showed increased N1a amplitudes, starting values and mean RT for validly relative to invalidly cued targets, and increased N1pc amplitudes for invalidly relative to validly cued targets. In ASD relative to NT, results showed a reduced modulation of N1a amplitudes for validly relative to invalidly cued targets, and an increased modulation of N1pc amplitudes for invalidly relative to validly cued targets. Increased N1pc amplitudes correlated with faster mean RT across groups. Against our expectations, N1a amplitudes were not correlated with starting values across groups and the cue validity effect on the perceptual decision bias and mean RT was not different between groups. However, increased N1a amplitudes correlated with higher starting values and faster mean RT in NT, but not in ASD.

Our findings of increased N1a amplitudes for validly cued targets as index of processing gain are in line with the neurophysiological model proposed by Feldman and Friston^12^. More specifically, the model by Feldman and Fristion^12^ predicts increased processing gain for targets presented at previously cued locations. One previous finding additionally reported increased anterior negativity within the N1 time window for validly cued targets^15^. The source of frontocentral negativity within the N1 time window has been attributed to the superior parietal cortex, near the intraparietal sulcus^52^. Scalp distributions in the present study, however, show a relatively more pronounced frontal distribution (see Supplementary Figs. S1–S3 online), which rather resembles a “prefrontal N1” (pN1) than a frontocentral N1 (N1a) component. Activity underlying the pN1 may originate from the anterior insula^15,53,54^. Interestingly, the insula is a highly-connected brain hub, which marks the most relevant incoming input for further processing depending on prior expectations^55^. Source-based analyses should further investigate whether a reduced modulation of early frontal negativity by perceptual expectations in ASD originates from reduced differential processing of validly and invalidly cued targets in the insula.

In the present study, findings across groups suggest an increased modulation of attention in response to invalidly cued targets, based on increased N1pc amplitudes for invalidly relative to validly cued targets. Several previous findings similarly reported an ‘attenuation-by-prediction’ effect on the N1pc amplitude^19,56^. One previous study instead reported an “increase-by-prediction” effect on the N1pc amplitude^17^. In this previous study, participants were instructed to detect a specific target feature and ignore another distractor feature throughout the task. Results showed increased N1pc amplitudes when target probability was higher. This suggests an effect in the opposite direction when perceptual expectations are manipulated probabilistically without an attentional cue varying on a trial-by-trial basis. According to hierarchical Bayesian models, attention increases when the estimated reliability of new incoming sensory input outweighs the estimated reliability of prior perceptual expectations^12,27^. An “increase-by-prediction” effect on the N1pc amplitude may thus reflect a higher reliance on sensory input with high as compared to low probability^17^. In Posner-like tasks, the “attenuation-by-prediction” effect may instead reflect more strongly that prior expectations across invalid trials are estimated as less reliable than those across valid trials. Both the “increase-by-prediction” and the “attenuation-by-prediction” effect on the N1pc may thus reflect increases in selective attention in a different experimental context. Activity underlying the N1pc would start in the early visual cortex, propagate towards higher areas in ventral extrastriate cortex and by about 160 ms recur in both the early visual cortex and the parietal cortex^57^. On a neural level, selective attention may manifest as a recurrent oscillatory process, with the N1pc and subsequent contralateral negativity each indexing separate periods^20^. Still, more research is needed to validate the N1pc amplitude as a specific correlate of selective attention. Future studies may investigate whether the N1pc amplitude specifically correlates with the estimated precision of prediction errors rather than with the unweighted prediction-error^58^.

Regarding the differential neural processes in ASD compared to NT, our results indicate a reduced differential processing of validly relative to invalidly cued targets and an increased attentional response to invalidly relative to validly cued targets. Our findings of a reduced modulation of the N1a amplitude by cue validity in ASD relative to NT replicate a previous finding based on a similar sample and paradigm^24^. EEG findings from other paradigms similarly suggest atypical sensory processing of unexpected sensory input in ASD^59,60^. As outlined above, a reduced modulation of early frontal negativity by perceptual expectations in ASD may arise from reduced differential processing of validly and invalidly cued targets in the insula. Previous findings indicate functional changes within the insula and atypical connectivity with the salience network in ASD^55^, a network which drives stimulus-driven re-orienting of attention^61^. Future research using source-based analyses may further examine whether sensory processing in the insula predicts atypical connectivity with the salience network in ASD. To the best of our knowledge, this is the first study to examine the N1pc as index of stimulus-driven selective attention in ASD. Previous evidence for an increased modulation of attention in ASD comes from a finding that showed increased modulation of pupil dilation during visuospatial orienting in ASD^8^. In contrast, other EEG-based findings reported a reduced attentional responsivity in ASD to novel click-sounds^62^ and to a rapid serial presentation of task-relevant and irrelevant stimuli^63^. These contrasting results may illustrate a reduced rather than increased modulation of attention in ASD in the presence of distracting visual information. That is, engaging visual input, such as watching a cartoon, may have disproportionally attenuated attention to novel click-sounds in children with ASD^62^. Furthermore, during a rapid, parallel presentation of target and distractor stimuli, reduced attentional responses in ASD were observed for both target and distractor stimuli^63^.

Despite the group differences in processing gain and attention, study findings do not clearly indicate how processing gain and attention influenced behavioral responses in participants with ASD. Our findings of faster mean RT and increased perceptual decision bias for validly relative to invalidly cued targets across groups are in line with previous literature^13^. In contrast with previous literature^3,24^, these effects were not different between groups, despite a statistical power to detect small-sized effects. In Posner-like paradigms, a predictive cue facilitates responses for a limited amount of time. That is, after about 300 ms, the facilitating effect of a predictive cue reverses and inhibits behavioral reaction times^64^. To measure the facilitating effects of predictive cues on target detection, the present study therefore used a short cue-target stimulus onset asynchrony (SOA) of 250 ms. Results of a meta-analysis on visuospatial orienting in ASD suggest that shorter SOA time are associated with increased group differences in mean RT^3^. Still, slower reaction times in ASD have been reported in comparable samples and based on Posner-like tasks with SOA times longer than 250 ms^8,24^. Those studies however used central, endogenous cues and distractor stimuli^8^ or an additional diagonal cue condition^24^, which makes the task more difficult. Taken together, this suggests a reduced difficulty level rather than SOA time may explain the null-findings on group differences in mean RT. Furthermore, in NT but not in ASD, we found large correlations between increased N1a amplitudes and faster mean RT and small correlations between increased N1a amplitudes and higher starting values. This suggests that, in contrast to ASD, in NT increased processing is strongly associated with faster mean RT and weakly associated with a higher perceptual decision bias. Re-analysis of the data without participants on stimulant medication (*n* = 3) furthermore revealed an additional small correlation between increased N1a amplitudes and lower rather than higher starting values in ASD. Acute effects of stimulants are associated with increased activation of the right inferior frontal cortex and the insula in patients with ADHD^51^. Stimulant use in the ASD group may thus have increased variability in early frontal activity captured by the N1a amplitude, but does not explain that increased processing differentially affected the perceptual decision bias and mean RT in ASD relative to NT. Still, whether reduced differential processing in ASD indeed results in a less precise perceptual decision bias should be further examined in studies controlling for the effect of stimulant medication. Regarding the influence of attention, increased N1pc amplitudes were moderately associated with faster mean RT across groups. Based on the 95% CI (see Sect. 3.2.5), results provide no convincing evidence for stronger correlations between increased N1pc amplitudes and faster mean RT in ASD. This argues against compensatory increases in attention influencing behavioral responses in ASD, though correlations within groups had reduced statistical power to detect medium-sized effects. Furthermore, in the present study, all outcome measures were aggregated across trials, which reduced the sensitivity to capture effects over time. During performance on a Posner task, changes in processing gain and attention are assumed to covary over time^12^. In contrast to less precise perceptual expectations in ASD, slower expectation updating in ASD predicts that changes in processing gain and attention differentially covary over time relative to NT. Such effects over time would also influence the correlations between the neurophysiological indices, perceptual decision bias and mean RT. Future studies may therefore analyze the effect of perceptual expectations in ASD using time-series data.

A final limitation was that central fixation to the target stimulus was not validated by an external method, such as eye-tracking. Participants that fail to maintain fixation may display additional frontal activity due to saccadic eye movements^65^. To minimize the influence of saccadic eye movements, saccadic eye movements were removed during EEG preprocessing using independent component analysis (ICA). Furthermore, after removing ICA components, segments with remaining artifacts such as eye movements or blinks were rejected and participants with more than one third of rejected trials were excluded from the analysis (NT: *N* = 1).

In conclusion, present results on neural correlates of perception and attention provide new evidence for an atypical anticipation of sensory input during visuospatial orienting in ASD. Results furthermore provide preliminary evidence that this atypical anticipation may differentially influence behavioral responses through perceptual decision bias in ASD as compared to TD. On a neural level, this atypical anticipation may attenuate differential sensory processing of unexpected and expected visual input, and underlie changes in stimulus-driven attention. Difficulties to adaptively adjust attention to incoming sensory input may negatively impact social communication skills in ASD throughout development^4^. Reduced differential processing in ASD may furthermore lead to more and more intense sensory experiences of surprise on a daily basis. Restricted, repetitive patterns of behavior in ASD, such as an increased insistence on sameness, may be a way to avoid or reduce such experiences as much as possible^66^. Nevertheless, those behaviors negatively impact learning and social adaptation throughout development^67^. Further research into the influence of perceptual expectations in ASD could therefore help to better understand what it means that individuals with ASD perceive the world differently, as well as the impact of these differences on other aspects of development.

## Supplementary Information

Supplementary Information 1.

Supplementary Figures.

Supplementary Tables.

## Data Availability

The datasets analyzed in the current study are available from the corresponding author on reasonable request.
