# Supplementary material for: The effect of perceptual expectation on processing gain, attention and the perceptual decision bias in children and adolescents with Autism Spectrum Disorder (ASD)
Source: Sci Rep. 2022 Dec 15;12:21688. doi: 10.1038/s41598-022-25971-z (PMC9755142; doi:10.1038/s41598-022-25971-z)
Supplement: Supplementary file 1 — Supplementary Information 1. [file 41598_2022_25971_MOESM1_ESM.docx]

**Perceptual expectations differentially modulate neural correlates of perception and attention in children and adolescents with Autism Spectrum Disorder**

Sara Boxhoorn*^1,^ Magdalena Schütz^1,^, Andreas M. Mühlherr^1^, Hannah Mössinger^1^, Christina Luckhardt^1,#^, Christine M. Freitag^1,#*^

^1^ Department of Child and Adolescent Psychiatry, Psychotherapy and Psychosomatics, University Hospital Frankfurt, Goethe University, Frankfurt am Main, Germany

^#^ joint last authors

**Supplementary information**

For each participant DDM parameters were estimated by fitting the same model using Fast-dm modelling technique and the Kolmogorov-Smirnov (KS) optimization procedure, which for each participant provided a fit-index *p* [1]. A Monte Carlo Simulation was run to obtain a reliable statistical threshold to evaluate model fit for each participant [2, 3]. 1000 random parameter sets were generated following the multivariate normal distribution based on the mean values and variance-covariance matrix of the estimated parameters using mvtnorm environment (*MASS* package; [4]). For each of those parameter-sets, one dataset was simulated using the construct-sample tool of fast-dm [1] (i.e. separately for each condition). Simulated data were subsequently fitted to the same diffusion model fitted to the empirical data of each participant. Results generated a distribution of 1000 p-values, and the 1% quantile of the distribution of p-values was selected as critical value (*p_c_* < 0.001) to evaluate model fit for each participant. Results for each participant showed significant model fit. In the table below descriptive information on the fit indices is provided for each group and the simulation study separately.

**Table 1**

*Mean fit indices for each group and the simulation study*

|  | ASD  (*n*=23) | | TD  (*n*=23) | | Simulation  (*n*=1000) | |
| --- | --- | --- | --- | --- | --- | --- |
|  | ***M*** | ***SD*** | ***M*** | ***SD*** | ***M*** | ***SD*** |
| **K-S fit (*p*)** | 0.893 | 0.135 | 0.856 | 0.133 | 0.913 | 0.14 |

**References**

1. Voss A, Voss. J. Fast-dm: a free program for efficient diffusion model analysis. *Behav. Res. Methods* **39,** 767-775; 10.3758/BF03192967 (2007).
2. Voss A, Nagler M, Lerche V. Diffusion models in experimental psychology: A practical introduction. Exp. Psychol. **60,** 385-402**;** [10.1027/1618-3169/a000218](https://doi.org/10.1027/1618-3169/a000218) (2013).
3. Voss A., Voss J., & Lerche V. Assessing cognitive processes with diffusion model analyses: a tutorial based on fast-dm-30. *Front. Psychol*. **6,** 336; 10.3389/fpsyg.2015.00336 (2015).
4. Venables WN, Ripley BD. *Modern Applied Statistics with S*. 4rth ed. (Springer, 2002). <http://www.stats.ox.ac.uk/pub/MASS4/> (2002).
