# Supplementary material for: The effect of perceptual expectation on processing gain, attention and the perceptual decision bias in children and adolescents with Autism Spectrum Disorder (ASD)
Source: Sci Rep. 2022 Dec 15;12:21688. doi: 10.1038/s41598-022-25971-z (PMC9755142; doi:10.1038/s41598-022-25971-z)

**Figure S1a**

*GA waveforms for midline- N1a electrode locations for the valid condition (solid line) and invalid condition (dashed line). Target onset at t=0. Blue shading: 130 – 180 ms.*


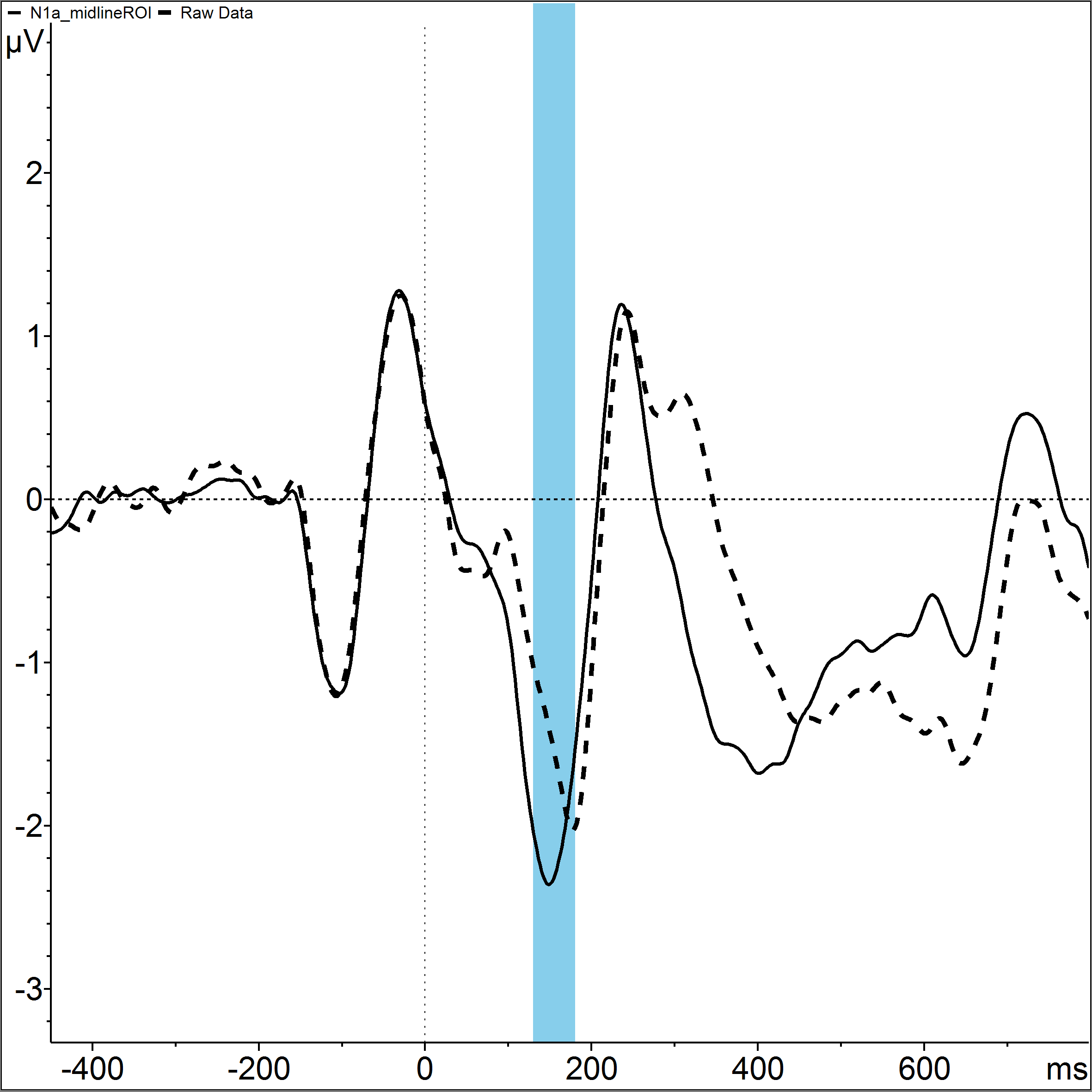


**Figure S1b***Corresponding scalp topographies for N1a components between 130 and 180 ms for the valid (upper image) and invalid condition (image below).*


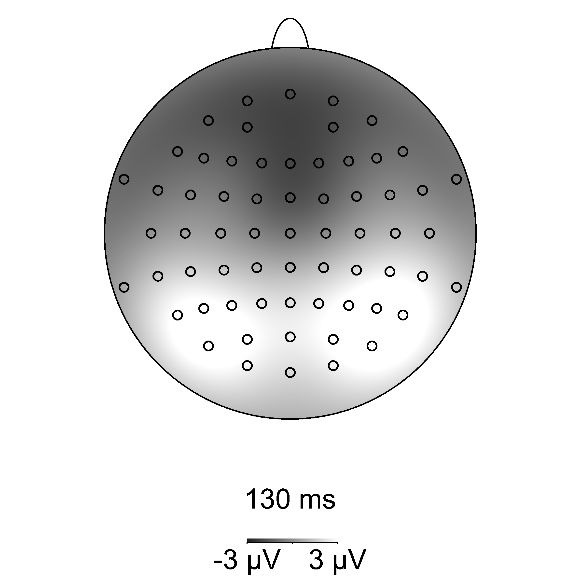


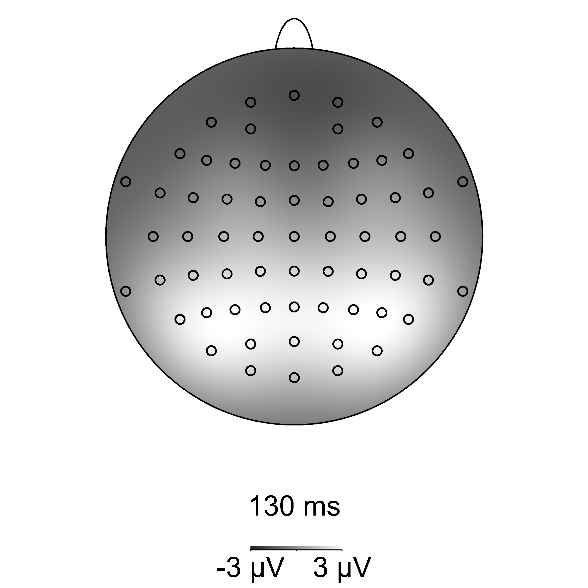


**Figure S2a**

*GA waveforms for midline- N1a electrode locations for the valid condition (solid line) and invalid condition (dashed line). Target onset at t=0. Blue shading: 130 – 180 ms.*


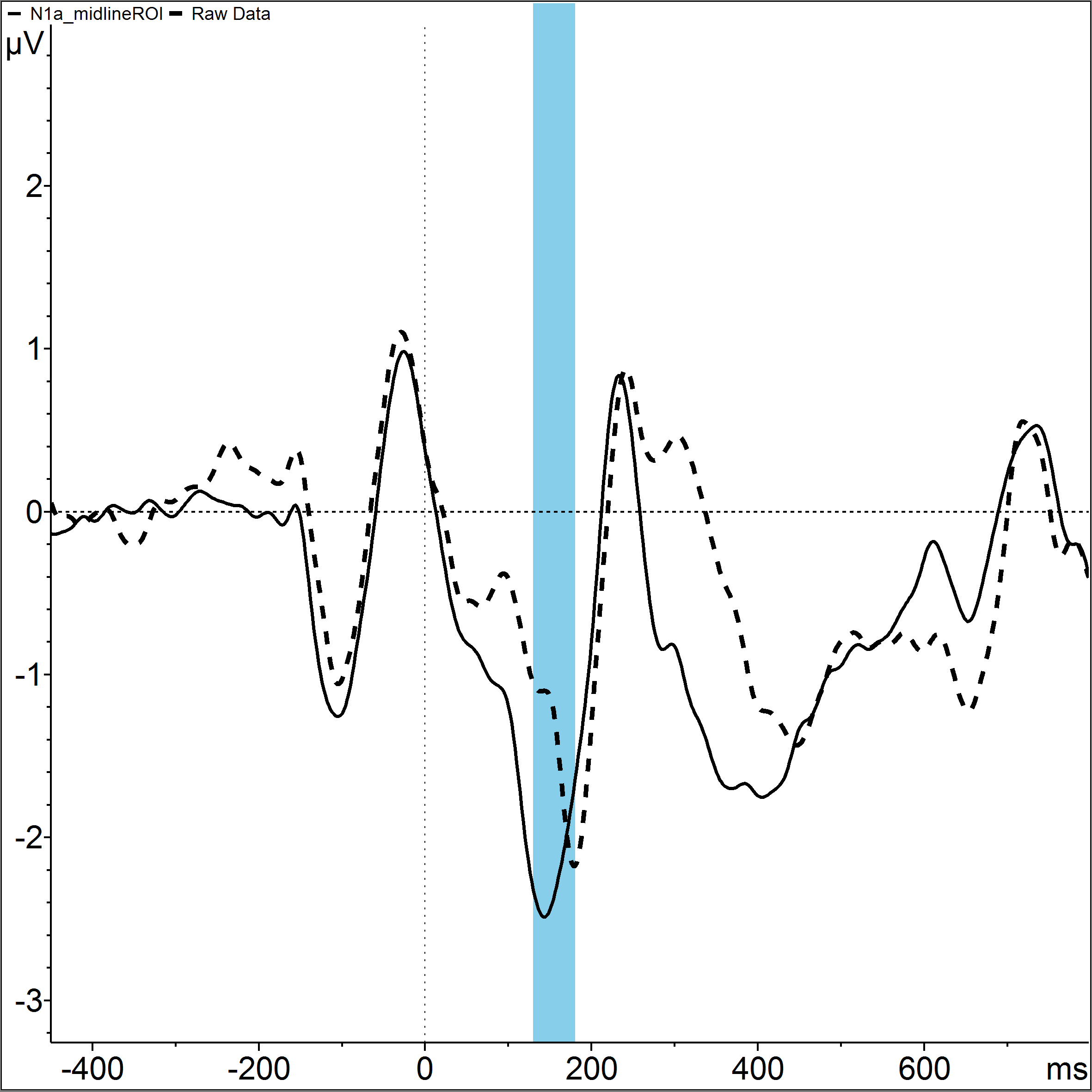


**Figure S2b**

*Corresponding scalp topographies for N1a components between 130 and 180 ms for the valid (upper image) and invalid condition (image below)*


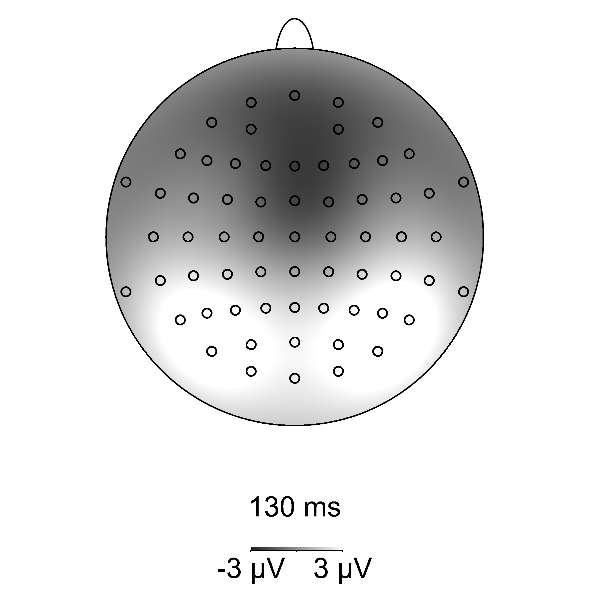


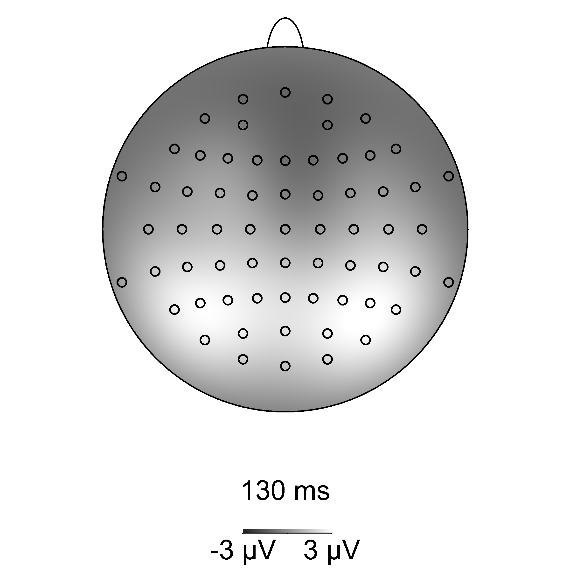


**Figure S3a**

*GA waveforms for midline- N1a electrode locations for the valid condition (solid line) and invalid condition (dashed line). Target onset at t=0. Blue shading: 130 – 180 ms.*


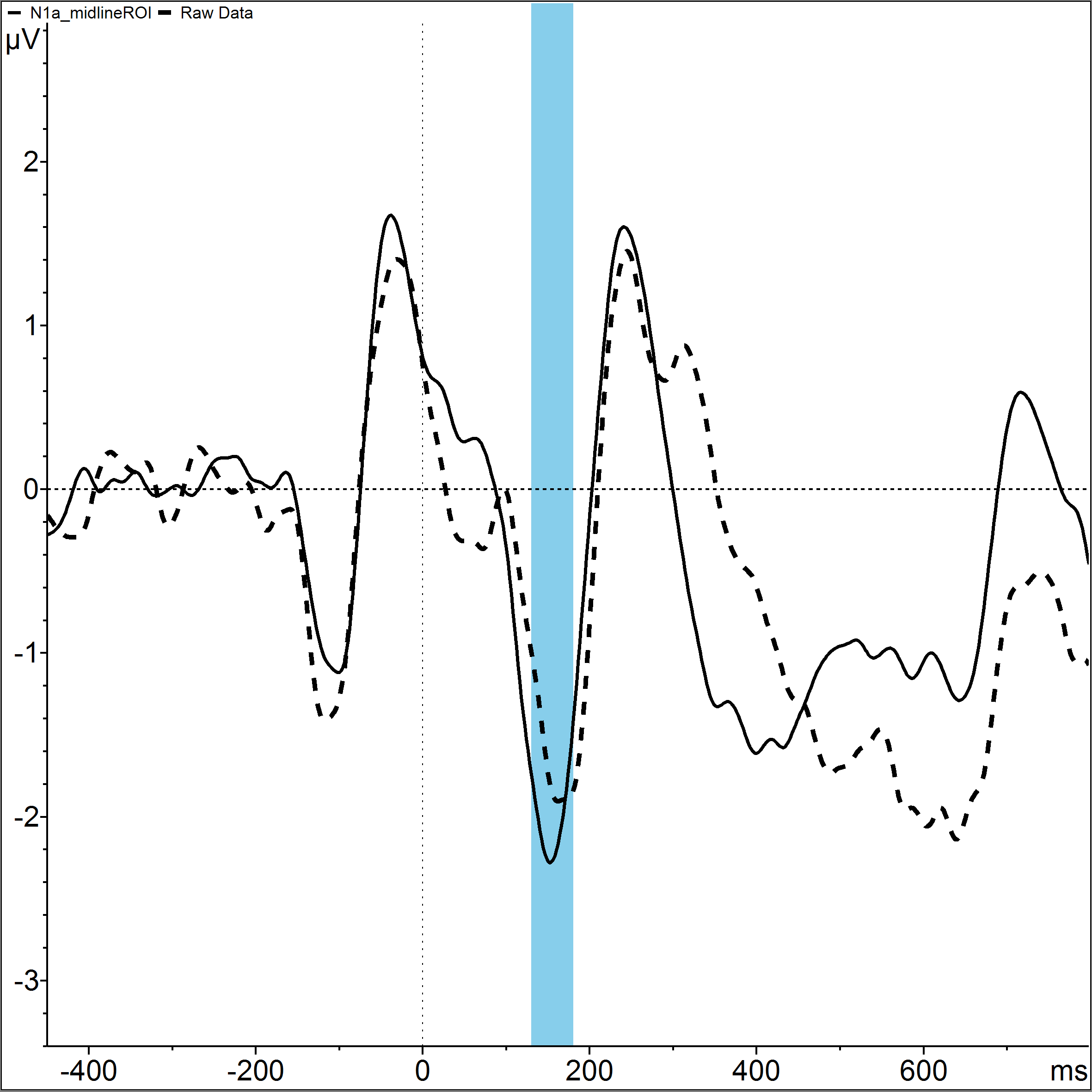


**Figure S3b**

*Corresponding scalp topographies for N1a components between 130 and 180 ms for the valid (upper image) and invalid condition (image below)*


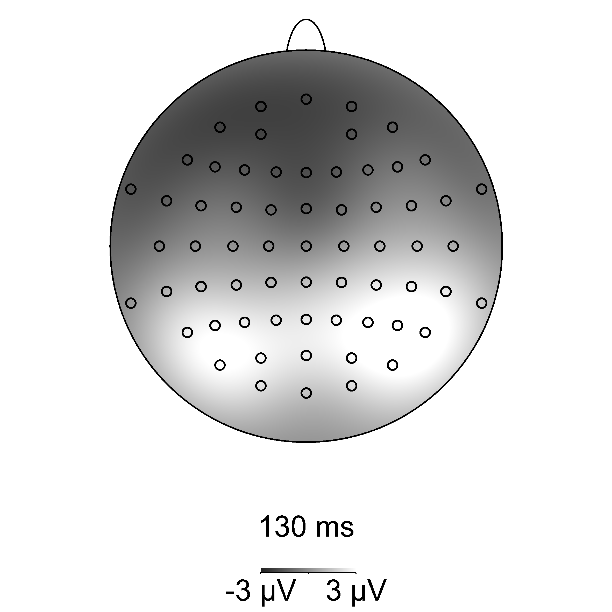

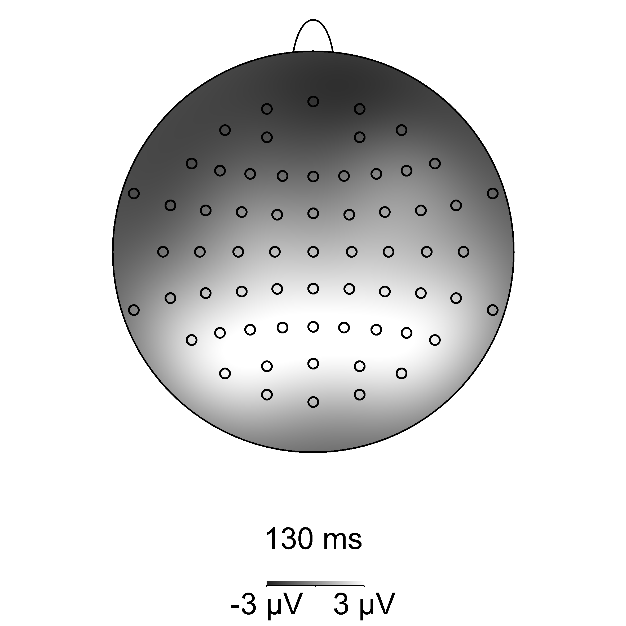


**Figure S4a**

*GA contralateral minus ipsilateral difference waves for the valid condition (solid line) and invalid condition (dashed line).Target onset at t=0. Blue shading: 160 – 200 ms.*


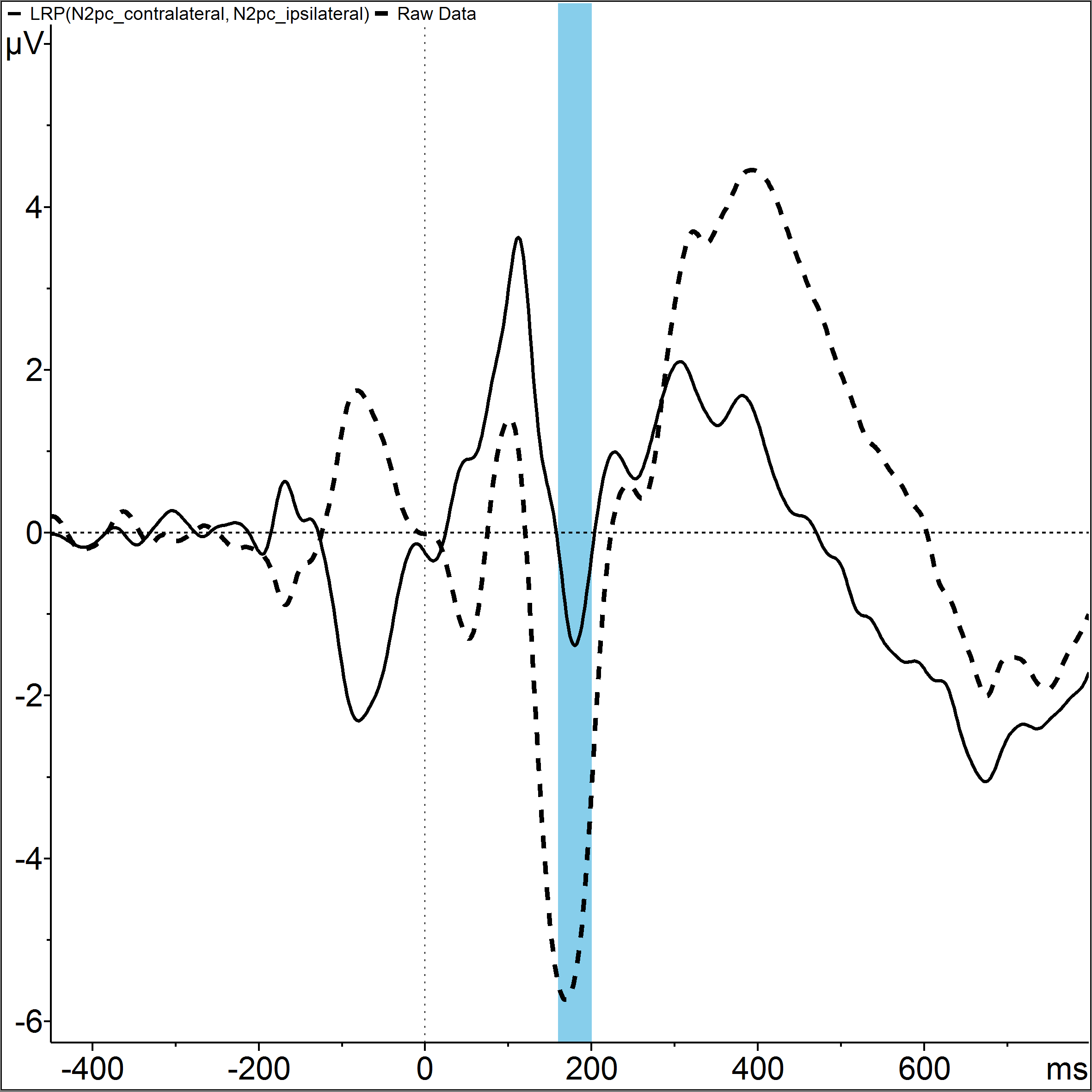


**Figure S4b**

*Corresponding scalp N1pc scalp topographies in the valid (160 - 190 ms; upper image) and invalid condition (170 - 200 ms; image below).*


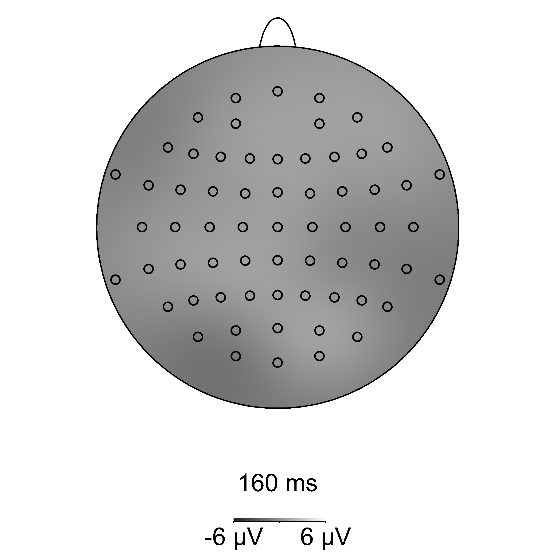


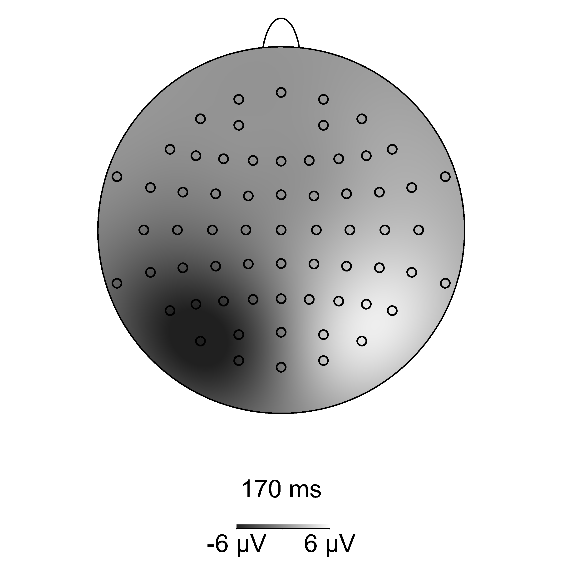


**Figure S5a**

*GA contralateral minus ipsilateral difference waves for the valid condition (solid line) and invalid condition (dashed line). Target onset at t=0. Blue shading: 160 – 200 ms.*


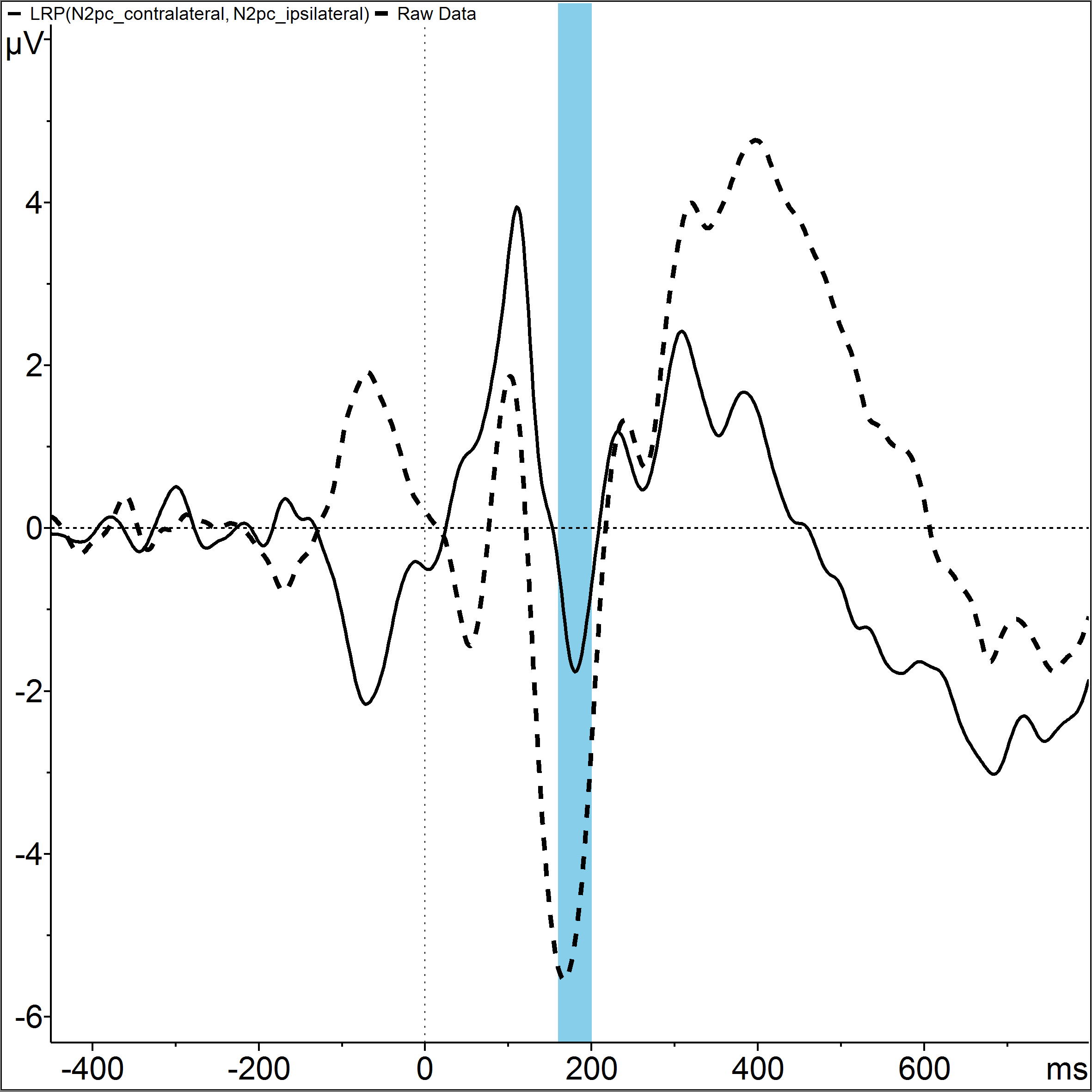


**Figure S5b**

*Corresponding scalp N1pc scalp topographies in the valid (160 - 190 ms; upper image) and invalid condition (170 - 200 ms; image below)*


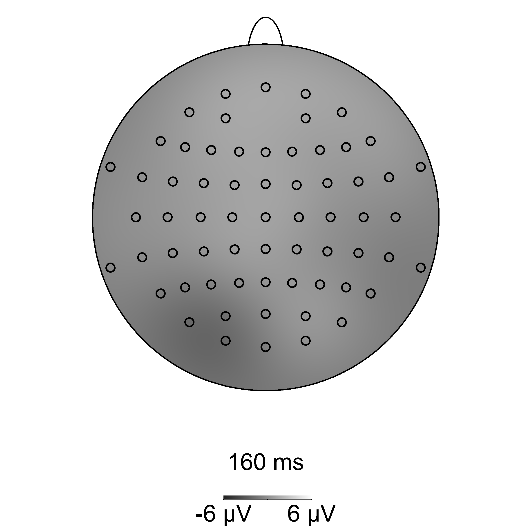

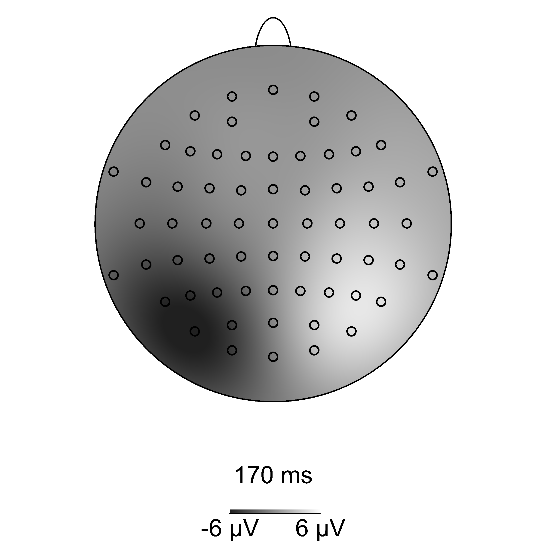


**Figure S6a**

*GA contralateral minus ipsilateral difference waves for the valid condition (solid line) and invalid condition (dashed line). Target onset at t=0. Blue shading: 160 – 200 ms.*


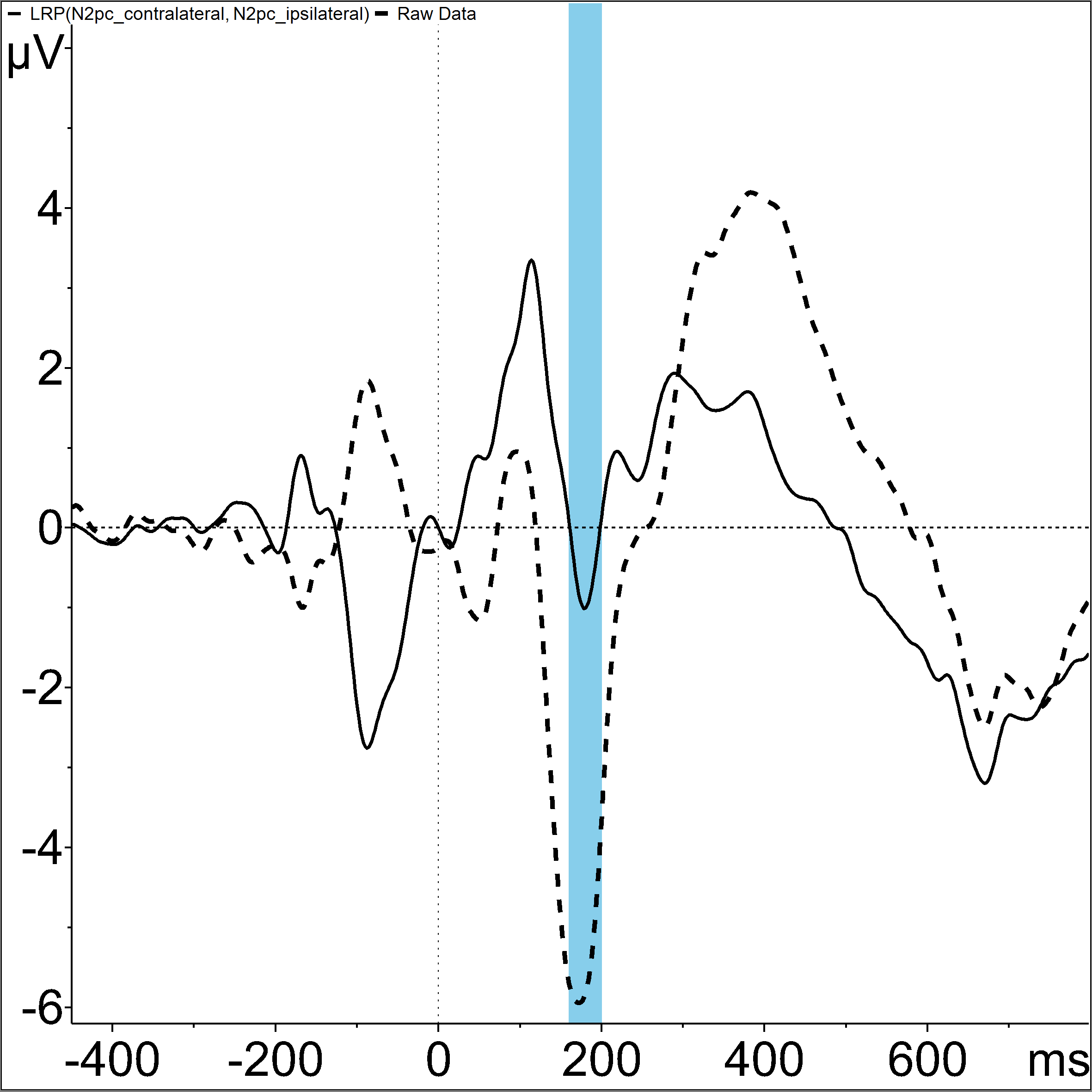


**Figure S6b**

*Corresponding scalp N1pc scalp topographies in the valid (160 - 190 ms; upper image) and invalid condition (170 - 200 ms; image below)*


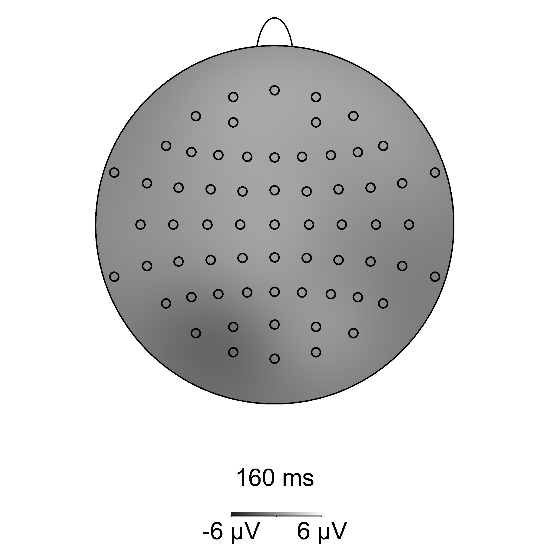


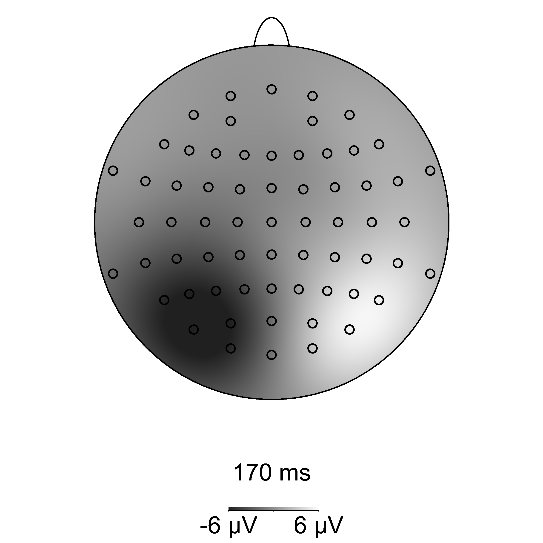

Supplement: Supplementary file 2 — Supplementary Figures. [file 41598_2022_25971_MOESM2_ESM.docx]
