# Supplementary material for: The effect of perceptual expectation on processing gain, attention and the perceptual decision bias in children and adolescents with Autism Spectrum Disorder (ASD)
Source: Sci Rep. 2022 Dec 15;12:21688. doi: 10.1038/s41598-022-25971-z (PMC9755142; doi:10.1038/s41598-022-25971-z)
Supplement: Supplementary file 3 — Supplementary Tables. [file 41598_2022_25971_MOESM3_ESM.docx]

| **Effect** |  |  |  |  |
| --- | --- | --- | --- | --- |
| **Main effects – Between/Across Subjects** | ***F* (1,38)** | ***p*** | **η_p_^2^** | **η^2^G** |
| group | 0.12 | 0.730 | 0.003 | 0.002 |
| ADHD symptom severity | 0.07 | 0.793 | 0.002 | 0.001 |
| age | 5.90 | 0.020 | 0.134 | 0.107 |
| **Main effects – Within Subjects** |  |  |  |  |
| cue validity | 4.92 | 0.033 | 0.115 | 0.016 |
|  | ***F* (1.72, 65.48)** |  |  |  |
| electrode location | 5.56 | 0.008 | 0.128 | 0.003 |
| **Interaction effects** | ***F* (1,38)** |  |  |  |
| group x cue validity | 4.04 | 0.051 | 0.096 | 0.013 |
| group x ADHD symptom severity | 0.13 | 0.725 | 0.003 | 0.002 |
| cue validity x  ADHD symptom severity | 3.23 | 0.080 | 0.075 | 0.010 |
| cue validity  x group x ADHD symptom severity | 4.81 | 0.034 | 0.112 | 0.016 |
|  | ***F* (1.72, 65.48)** |  |  |  |
| group x electrode location | 2.42 | 0.104 | 0.060 | 0.001 |
| ADHD symptom severity x electrode location | 1.10 | 0.332 | 0.028 | <0.001 |
| group x ADHD symptom severity x electrode location | 0.48 | 0.592 | 0.013 | <.001 |
|  | ***F* (1.75, 66.63)** |  |  |  |
| electrode location x cue validity | 0.75 | 0.458 | 0.019 | <.001 |
| group x cue validity  x electrode location | 4.50 | 0.018 | 0.106 | 0.001 |
| ADHD symptom severity x cue validity  x electrode location | 6.46 | 0.002 | 0.145 | 0.002 |
| group x cue validity  x electrode location x ADHD symptom severity | 1.63 | 0.206 | 0.041 | <0.001 |

**Table S1**

*The cue validity effect (valid cue vs. invalid cue) and group differences in the cue validity effect on N1a amplitude without participants with ASD on stimulant medication (N = 3)*

*Legend.* **ADHD**: Attention-Deficit/Hyperactivity Disorder

| **Effect** |  |  |  |  |
| --- | --- | --- | --- | --- |
| **Main effects – Between Subjects** | ***F* (1,41)** | ***p*** | **η_p_^2^** | **η^2^G** |
| group | 1.01 | 0.322 | 0.024 | 0.012 |
| ADHD symptom severity | 0.03 | 0.859 | <0.001 | <0.001 |
| age | 0.43 | 0.515 | 0.010 | 0.005 |
| **Main effects – Within Subjects** |  |  |  |  |
| cue validity | 2.24 | 0.142 | 0.012 | 0.052 |
|  | ***F* (1.78, 72.90)** |  |  |  |
| electrode location | 0.21 | 0.786 | 0.005 | <0.001 |
| **Interaction effects** | ***F* (1,41)** |  |  |  |
| group x cue validity | 2.00 | 0.165 | 0.046 | 0.010 |
| group x ADHD symptom severity | 0.01 | 0.907 | < 0.001 | < 0.001 |
| cue validity x  ADHD symptom severity | 4.02 | 0.051 | 0.089 | 0.021 |
| perceptual expectation x group x ADHD symptom severity | 4.76 | 0.035 | 0.104 | 0.025 |
|  | ***F* (1.78, 72.90)** |  |  |  |
| group x electrode location | 0.31 | 0.707 | 0.008 | <0.001 |
| ADHD symptom severity x electrode location | 0.26 | 0.750 | 0.006 | <.001 |
| group x ADHD symptom severity x electrode location | 0.35 | 0.680 | 0.008 | <.001 |
|  | ***F* (1.82, 74.62)** |  |  |  |
| electrode location x cue validity | 1.39 | 0.254 | 0.033 | 0.004 |
| group x cue validity x electrode location | 0.44 | 0.629 | 0.011 | 0.001 |
| ADHD symptom severity x cue validity x electrode location | 0.47 | 0.607 | 0.011 | 0.001 |
| group x cue validity x electrode location x ADHD symptom severity | 0.51 | 0.585 | 0.012 | 0.001 |

**Table S2** *Cue validity effect (invalid cue vs. valid cue) and group differences in the cue validity effect on 50% fractional peak N1a onset latency*

*Legend.* **ADHD**: Attention-Deficit/Hyperactivity Disorder

**Table S3**

*The cue validity effect (valid cue vs. invalid) and group differences in the cue validity effect on the N1pc amplitude without participants with ASD on stimulant medication (N = 3)*

| **Effect** |  |  |  |  |
| --- | --- | --- | --- | --- |
| **Main effects – Between/Across Subjects** | ***F* (1,38)** | ***p*** | **η_p_^2^** | **η^2^G** |
| group | 0.14 | 0.709 | 0.004 | 0.003 |
| ADHD symptom severity | 0.66 | 0.422 | 0.017 | 0.013 |
| age | 1.51 | 0.227 | 0.038 | 0.029 |
| **Main effect – Within Subjects** |  |  |  |  |
| cue validity | 24.03 | < 0.001 | 0.387 | 0.0797 |
| **Interaction effects** |  |  |  |  |
| group x cue validity | 6.26 | 0.017 | 0.142 | 0.027 |
| group x ADHD symptom severity | 0.00 | 0.995 | <0.001 | <0.001 |
| ADHD symptom severity x cue validity | 2.35 | 0.134 | 0.058 | 0.010 |
| group x cue validity x ADHD symptom severity | 1.79 | 0.189 | 0.045 | 0.008 |

*Legend.* **ADHD**: Attention-Deficit/Hyperactivity Disorder

**Table S4**

*Cue validity effect (invalid cue vs. valid cue) and group differences in the cue validity effect on 50% fractional peak N1pc onset latency*

| **Effect** |  |  |  |  |
| --- | --- | --- | --- | --- |
| **Main effect – Between Subjects** | ***F* (1,41)** | ***p*** | **η_p_^2^** | **η^2^G** |
| group | 0.09 | 0.767 | 0.002 | <0.001 |
| ADHD symptom severity | 0.28 | 0.602 | 0.007 | 0.003 |
| age | 0.31 | 0.580 | 0.008 | 0.003 |
| **Main effect –**  **Within Subjects** |  |  |  |  |
| cue validity | 0.90 | 0.348 | 0.022 | 0.011 |
| **Interaction effects** |  |  |  |  |
| group x cue validity | 0.22 | 0.644 | 0.005 | 0.003 |
| group x ADHD symptom severity | 0.09 | 0.770 | 0.002 | <.001 |
| cue validity x ADHD symptom severity | 0.54 | 0.466 | 0.013 | 0.007 |
| group x cue validity x ADHD symptom severity | 0.47 | 0.497 | 0.011 | 0.006 |

*Legend.* **ADHD**: Attention-Deficit/Hyperactivity Disorder
